# Involvement of STAT4 in IgG subtype switching and ocular HSV-1 replication in mice

**Published:** 2010-01-26

**Authors:** Sariah J. Allen, Kevin R. Mott, Homayon Ghiasi

**Affiliations:** Center for Neurobiology and Vaccine Development, Ophthalmology Research Laboratories, Department of Surgery, CSMC Burns & Allen Research Institute, Los Angeles, CA

## Abstract

**Purpose:**

To assess the relative impact of elevated T-helper 2 (T_H_2)- and reduced T-Helper 1 (T_H_1)-dependent immune responses on ocular herpes simplex virus type 1 (HSV-1) infection.

**Methods:**

Signal transducer and activator of transcription protein 4 knockout mice (BALB/c-STAT4^−/−^) and wild-type BALB/c control mice were immunized with avirulent HSV-1 strain KOS or were mock-immunized. Three weeks after the third immunization, neutralizing antibody titers were determined by plaque reduction assays. Following ocular infection with virulent HSV-1 strain McKrae, viral replication in the eye, blepharitis, corneal scarring (CS), survival, and immunoglobulin (Ig) isotypes in sera were determined.

**Results:**

Vaccinated STAT4^−/−^ and BALB/c mice contained significant and similar neutralizing antibody titers and were completely protected against HSV-1-induced death and CS. In contrast to vaccinated STAT4^−/−^ mice, mock-vaccinated STAT4^−/−^ mice had higher ocular HSV-1 titers than mock-vaccinated BALB/c mice on days 2–3 post-ocular infection. There were also significant differences in the levels of IgG2a, IgG2b, and IgG3 in the sera of STAT4^−/−^ mice when compared to the control BALB/c mice.

**Conclusions:**

These results suggest that the absence of T_H_1 cytokine responses did alter protection against viral replication and IgG isotypes but not eye disease or survival.

## Introduction

Signal transducers and activators of transcription (STAT) proteins are activated in response to a large number of cytokines, growth factors, and hormones [1]. Upon activation following the binding of ligands to their receptors, STAT proteins dimerize, translocate to the nucleus, and bind to the promoters of specific target genes. At present the STAT family is classified into seven groups [2] of cytoplasmic proteins, which are activated by phosphorylation of a specific tyrosine [3]. Although some cytokines and growth factors can activate multiple STAT proteins, certain STAT proteins are activated with considerable specificity. In turn, each activated STAT protein activates transcription of a specific cytokine. For example, STAT6 is involved in production of several interleukins (IL) such as IL-4 and IL-13 [4,5], while STAT4 is involved in production of IL-2 [6,7]. Thus, STAT6^−/−^ mice have a reduced T-helper 2 (T_H_2)-mediated immune response, while STAT4^−/−^ mice have an increased T_H_2-mediated immune response.

Following stimulation by foreign antigens, CD4**^+^** and CD8**^+^** T-cell clones of mice and humans produce specific patterns of cytokine expression [8,9]. Based on the cytokines produced, CD4**^+^** T cells are designated T_H_1 or T_H_2, and CD8**^+^** T cells are designated T_C_1 or T_C_2 [8,10,11]. Usually, either a T_H_1/T_C_1 or a T_H_2/T_C_2 cytokine pattern predominates in response to a specific antigenic challenge [12-14]. T_H_1/T_C_1 cells are involved in cellular immunity (delayed type hypersensitivity and cellular cytotoxicity) and produce IL-2, tumor necrosis factor beta (TNF-β), and interferon-gamma (IFN-γ). T_H_2/T_C_2 cells are involved in humoral immunity (antibody mediated) and produce IL-4, IL-5, IL-6, and IL-10 [9,15]. IL-4 enhances T_H_2/T_C_2 development and inhibits T_H_1/T_C_1 development [16,17]. IL-2 stimulates development of T_H_1/T_C_1 and inhibits development of T_H_2/T_C_2 [18,19]. The T_H_1/T_C_1 to T_H_2/T_C_2 balance determines the outcome of a wide variety of immune responses involving infectious, autoimmune, and allergic diseases [10].

We previously demonstrated faster clearance and lower eye disease in STAT6^−/−^ mice [20]. These results indicated that increased level of IL-2 in STAT6^−/−^ mice was associated with improved vaccine efficacy. Immunohistochemical analyses of corneal sections of ocularly infected mice had shown that lack of protection against corneal scarring (CS) correlated with the absence of neutralizing antibody titer and the presence of IL-4 in the cornea [13,21]. Since IL-4 is an indicator of a T_H_2 response [8,14], these results suggested that T_H_2 responses are either neutral or enhance CS [13,22]. The studies presented here with STAT4^−/−^ mice, which are deficient in IL-2 production and lack a T_H_1 response, were undertaken to determine if these observed correlations reflected function. We report that the absence of T_H_1 and elevation of T_H_2 responses in STAT4^−/−^ mice had no role in protection against ocular herpes simpex virus type 1 (HSV-1) infection but did have an effect on immunoglobulin-G (IgG)-subtype switching and early viral replication.

## Methods

### Virus and cells

Plaque-purified HSV-1 strains (maintained in-house) were grown in rabbit skin (RS) cell monolayers in minimal essential media (MEM) containing 5% fetal bovine serum. McKrae, a stromal disease-causing neurovirulent HSV-1 strain was the ocular challenge virus. KOS, a avirulent nonstromal disease-producing strain was used as a live virus vaccine.

### Mice

All animal procedures adhered to the Association for Research in Vision and Ophthalmology (ARVO) statement for the Use of Animals in Ophthalmic and Vision Research and according to institutional animal care and use guidelines. Six-week-old inbred BALB/c mice and homozygous BALB/c-STAT4^−/−^ mice (Jackson Laboratory, Bar Harbor, ME) were used in this study.

### Vaccinations of mice

Mice were vaccinated three times intraperitoneum (IP) at 3-week intervals with 2×10^5^ plaque-forming units (PFU) of live KOS in tissue culture media. Mock-vaccinated mice were similarly inoculated but with tissue culture media (MEM with %5 FBS) alone. Serum-neutralizing antibody titers were determined by 50% plaque reduction assays, as we described previously [23], using sera collected 3 weeks after the final vaccination. Briefly, the sera from vaccinated or mock-vaccinated mice were heat inactivated for 30 min. at 56 °C, diluted in MEM, mixed with 200 PFU of HSV-1 strain McKrae, and incubated for 30 min at 37 °C.  Samples were added to RS cells in 6-well microtiter plates, the plates were incubated at 37 °C for 72 h, stained with 1% crystal violet, and the plaques were counted.  The means of the antibody titers (50% plaque reduction) were expressed as the reciprocal of the serum dilution.

### Ocular infection

Mice were infected ocularly, without corneal scarification, with 2×10^5^ PFU of HSV-1 strain McKrae per eye, in 1 μl of tissue culture medium [23].

### Titration of virus in tears

Tear films were collected from both eyes of five or ten mice per group at various times, using a Dacron-tipped swab [22]. Each swab was placed in 0.5 ml tissue culture medium, squeezed, and the amount of virus was determined by a standard plaque assay on RS cells.

### Analysis of immunoglobulin subtypes and isotypes in the sera

Mice were bled by retro-orbital bleeding; sera were collected, and stored at -80 °C until use. IgG1, IgG2a, IgG2b, IgG3, IgM, and IgA concentrations were determined in sera collected from vaccinated mice, using a mouse immunoglobulin isotyping enzyme-linked immunosorbent assay (ELISA) kit (Becton Dickinson, San Diego, CA). Briefly, plates were coated with different rat anti-mouse antibody isotypes and incubated at 4 °C overnight. Sera was then added to each well, incubated overnight at 4 °C and washed with 0.05% Tween in 1× phosphate-buffered saline (PBS). Horseradish peroxidase (HRP)-labeled rat anti-mouse immunoglobulin G (IgG) was incubated for 1 h at room temperature and color was developed using an enzyme substrate solution (kit provided) for 10 min at room temperature.  Concentration was determined by spectrophotometric absorbance at 450 nm.

### Monitoring blepharitis and corneal scarring

The severity of blepharitis and CS were scored in a masked fashion by examination with slit lamp biomicroscope following addition of 1% fluorescein as eye drops. Disease was scored on a 0 to 4 scale (0=no disease, 1=25%, 2=50%, 3=75%, and 4=100% involvement) as we described previously [24].

### Statistical analysis

Protective parameters were analyzed by the Student *t* test and Fisher's exact test, using Instat (GraphPad, San Diego, CA). Results were considered statistically significant when the p value was <0.05.

## Results

### Herpes simplex virus type 1 (HSV-1) neutralizing antibody

**Figure 1:**
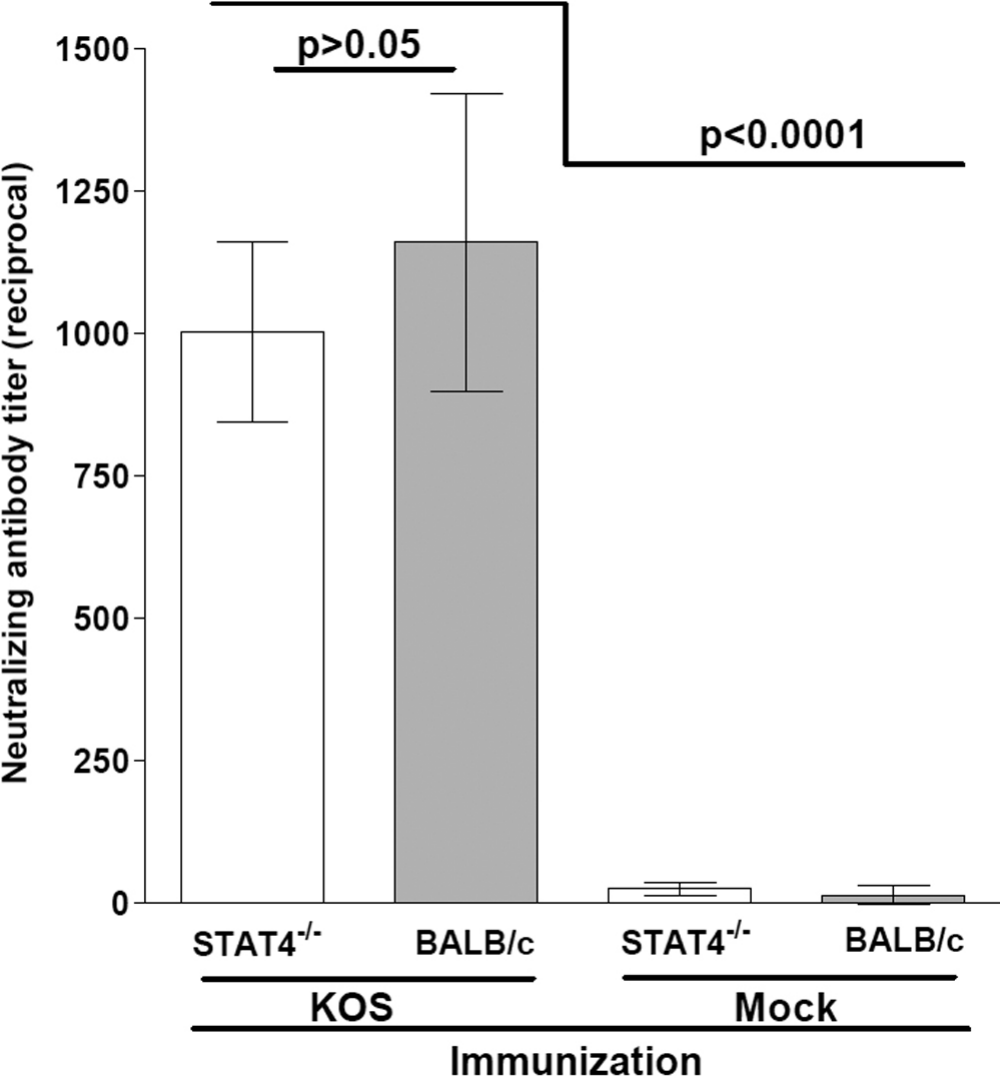
Neutralizing antibody titers in vaccinated mice. Mice were vaccinated three times and bled 3 weeks after the third vaccination. Neutralization titers are expressed as the reciprocal of the geometric means± the standard error of the mean (SEM) from seven individual mice sera. The p values were calculated from the Student *t* test.

BALB/c-STAT4^−/−^ and wild-type BALB/c mice were vaccinated three times with an avirulent HSV-1 strain KOS in MEM or mock-vaccinated with MEM alone, as described in the Methods section. Three weeks after the third vaccination, sera were collected and individually heat inactivated. Neutralization titers of these sera were then determined by plaque reduction assays. The average neutralizing antibody titer for vaccinated STAT4^−/−^ mice was not significantly different than for vaccinated BALB/c mice (Figure 1; p>0.05, Student *t* test). As expected, both vaccinated groups had significantly higher neutralizing antibody titers than did the mock-vaccinated mice (Figure 1; p<0.0001, Student *t* test).

### Immunoglobulin isotypes and subtypes

Sera from vaccinated mice were analyzed for the relative expression of each Ig subtype and IgG subtypes (Table 1). No significant differences were detected between the two groups for IgG1, IgA, or IgM. However, we did detect a significant decrease in the levels of IgG2a, IgG2b, and IgG3 in the STAT4^−/−^ mice. Thus, the absence of STAT4 altered the IgG2a, IgG2b, and IgG3 subtypes in vaccinated mice when compared with mock-vaccinated control wild-type mice.

**Table 1:** Immunoglobulin isotypes in the blood of vaccinated STAT4^−/−^ and BALB/c mice.

**Ig isotype**	**STAT4^−/−^**	**BALB/c**	**p**
IgG1	2.34±0.07	2.44±0.02	0.2068
IgG2a	2.04±0.14	2.62±0.09	0.0083
IgG2b	2.00±0.07	2.32±0.07	0.0120
IgG3	1.21±0.03	1.49±0.10	0.0278
IgA	1.98±0.17	2.31±0.05	0.0996
IgM	1.22±0.06	1.47±0.11	0.0811

Optical density (OD) of immunoglobulin isotypes and subtypes were determined (as described in Methods) in sera collected from five vaccinated STAT4^−/−^ or vaccinated BALB/c mice three weeks after the third vaccination and before ocular infection. Student’s *t*-test was used to compare results from vaccinated STAT4^−/−^ mice to vaccinated BALB/c mice.

### Vaccine protection against lethal ocular infection

Vaccinated and mock-vaccinated STAT4^−/−^and BALB/c mice were infected ocularly with 2×10^5^ PFU/eye of HSV-1 strain McKrae, as described in the Methods section. All of the vaccinated STAT4^−/−^ and BALB/c mice survived lethal ocular infection (Table 2). The vaccine-induced protection was highly significant for both groups compared to their mock-vaccinated counterparts (Table 2). Thus, even in the absence of STAT4, vaccination protected 100% of the mice against lethal ocular infection.

**Table 2:** Survival following ocular HSV-1 infection of vaccinated mice.

** Vaccine**	**Survival/Total**	
	**STAT4^−/−^**	**BALB/C**
KOS	10/10 (100%)	10/10 (100%)
Mock	3/10 (30%)	2/20 (20%)
p (KOS versus Mock)	0.0031	0.0007

Mice were vaccinated three times and infected ocularly with McKrae as described in the Methods. p-Values were determined using Fisher’s exact test.

### Herpes simplex virus type 1 (HSV-1) ocular clearance in STAT4^−/−^ mice

**Figure 2:**
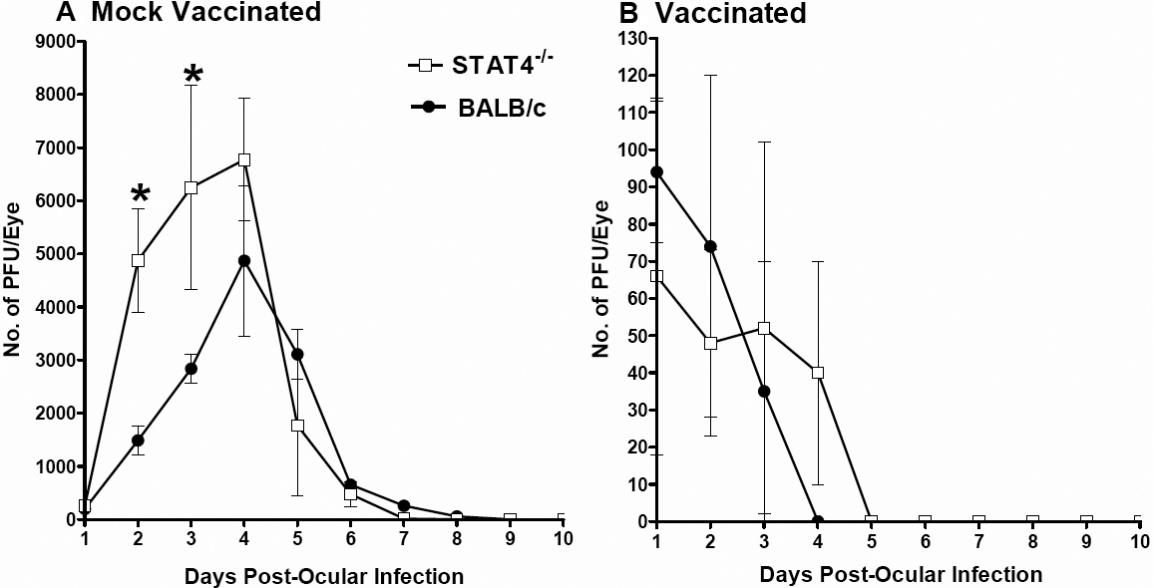
Virus titers in mouse eyes following ocular infection. Three weeks after the third vaccination, STAT4 knockout (STAT4^−/−^) (open symbols) and BALB/c mice (closed symbols) were infected ocularly, and the presence of Herpes Simplex Virus Type 1 (HSV-1) in tear films was monitored daily. For each point, the virus titer (y-axis) represents the average of the titers from 20 eyes. The error bars indicate the standard errors. **A**: Graph A shows virus replication in mock-vaccinated mice infected with 2×10^5^ plaque-forming units (PFU) per eye. **B**: Graph B shows virus replication in KOS-vaccinated mice infected with 2×10^5^ PFU/eye. Asterisks indicate significance (p<0.05) when titers are compared with the Student t test.

Tear films from mice infected ocularly with 2×10^5^ PFU/eye of HSV-1 (strain McKrae) were collected from 20 eyes/group on days 1–10 post infection (PI), and the amount of infectious HSV-1 was determined (Figure 2A,B). Mock-vaccinated STAT4^−/−^ mice had no significant differences in ocular HSV-1 titers on days 1 and on days 4 thru 10 PI (Figure 2A; p>0.05), while STAT4^−/−^ mice had significantly higher virus titers during days 2–3 PI than their corresponding BALB/c mice (Figure 2A; p<0.05); by day 4 PI, no differences were detected between STAT4^−/−^ mice and BALB/c mice. No significant differences were detected between virus titers in vaccinated STAT4^−/−^ mice or their corresponding BALB/c control (Figure 2B; p>0.05). However, in vaccinated STAT4^−/−^ mice, virus was completely cleared by day 5 PI (Figure 2B). In contrast, in vaccinated BALB/c mice, HSV-1 was completely cleared by day 4 PI (Figure 2B). Thus, during early times PI in mock-vaccinated but not vaccinated mice, the absence of STAT4 appears to enhance HSV-1 replication and in vaccinated mice STAT4^−/−^ leads to a slightly longer time to viral clearance.

### Protection against blepharitis and corneal scarring

**Figure 3:**
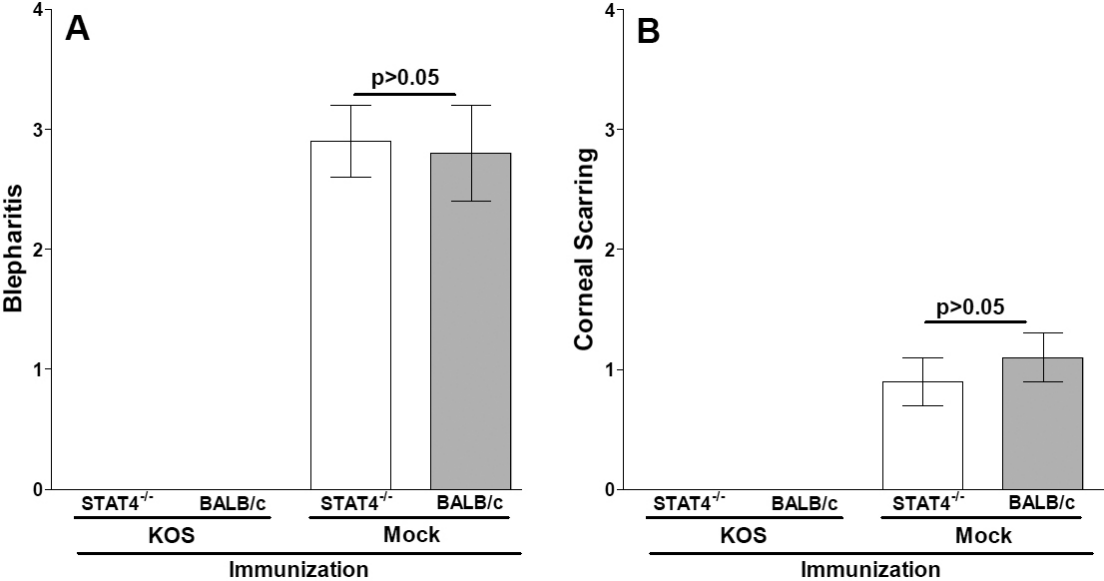
Blepharitis and corneal scarring (CS) following ocular infection. Three weeks after the third vaccination, STAT4^−/−^ and BALB/c mice were infected infected ocularly with Herpes simplex virus type 1 neurovirulent strain McKrae. Blepharitis was measured 7 days after ocular infection based on 20 eyes, while CS was measured 28 days after ocular infection from surviving mice listed in Table 2. The error bars indicate the standard errors. **A**: Graph A shows blepharitis on day 7 post-infection. **B**: Graph B shows CS on day 28 post-infection. The p values were calculated from the Student *t* test.

Herpetic blepharitis was measured 7 days after ocular infection, as described in the Methods section. Vaccinated STAT4^−/−^ and BALB/c mice were completely protected against blepharitis (Figure 3A; KOS, p>0.05). Mock-vaccinated STAT4^−/−^ and BALB/c mice exhibited similar levels of blepharitis, and both groups had significantly higher levels of blepharitis than did their vaccinated counterparts (Figure 3A; p<0.0001). CS was measured in surviving mice on day 28 post-ocular infection, as described in the Methods section. All of the vaccinated STAT4^−/−^ and BALB/c mice were completely protected against CS (Figure 3B; KOS, p>0.05). The protection against CS in vaccinated mice was highly significant when compared with their corresponding mock-vaccinated mice (Figure 3B; p<0.0001, Student *t* test). The level of CS between mock-vaccinated STAT4^−/−^ and BALB/c mice was similar (Figure 3B; Mock, p>0.05). Thus, the absence of STAT4 did not alter the level of blepharitis or CS in either vaccinated or mock-vaccinated mice.

## Discussion

HSV-1 infections are among the most frequent serious viral eye infections in the USA and are a major cause of viral-induced blindness [24-29]. HSV-1-induced CS, also broadly referred to as herpes stromal keratitis, can lead to blindness, and HSV-1 is the leading cause of corneal blindness from an infectious agent in developed countries [28,30,31]. The immune response(s) leading to CS following ocular HSV-1 infection is a T-helper response, and both T_H_1 and T_H_2 have been implicated [13,32,33]. Previous work demonstrated that an increase of T_H_1 response leads to lesser herpetic eye disease, an increase of neutralizing antibody titers, and ultimately an expedited viral clearance in STAT6^−/−^ knockout mice [20].

The present study was designed to extend our previous study with STAT6^−/−^ mice to determine what role, if any, elevation of T_H_2- and reduction of T_H_1-mediated immune responses may play in viral clearance and eye disease, using STAT4^−/−^ mice. The STAT4^−/−^ mice have impaired IL-12 processes and thus a reduced T_H_1 response and an increased T_H_2 response [34,35]. One would expect worsened eye disease in these animals, as IL-4 has been previously correlated with enhanced eye disease and increased HSV-1 replication [36,37]. However, we observed equal levels of eye disease, equal survival, and generally equal viral replication between STAT4^−/−^ and wild-type control BALB/c mice. Also, STAT4^−/−^ did not affect vaccine-induced protection as survival, eye disease, and viral replication were equal between the immunized STAT4^−/−^ mice and the immunized BALB/c control mice.

At the antibody level as we have shown here, the T_H_2 responses are associated with greater production of IgG1 and IgG2b, while the T_H_1 responses are associated with production of the IgG2a and IgG3 antibody (Ab) subclasses [38-40]. We observed a highly significant decrease in the level of IgG2a in the sera of STAT4^−/−^ mice when compared to BALB/c control mice and to our published results with STAT6^−/−^ mice [20]. This is most likely caused by the reduction in IFN-γ, as this cytokine has been shown to stimulate IgG2a secretion [41], and a reduction in IFN-γ production has been reported in STAT4^−/−^ mice [42]. Since T_H_1 cells are associated with the production of IgG2a antibodies [43], our results of decreased IgG2a levels correlate with a reduced T_H_1 response in the STAT4^−/−^ vaccinated mice. It was shown previously that IgG2a antibodies are associated with efficacious viral vaccines [43]. The decrease in expression of IgG2a in STAT4^−/−^ vaccinated mice is associated with a slight reduction in vaccine efficacy. Since IgG2a is still produced, most likely due to the immunization response, there is still protection. However, this might explain the increased early viral replication seen in STAT4^−/−^ mock-vaccinated mice as well as the delayed viral clearance observed in STAT4^−/−^ vaccinated mice. Murine-neutralizing antibody against HSV-1 is predominantly associated with IgG2a [44-46]. Previous studies have demonstrated that human IgG1, IgG3, and IgG4 all have neutralizing ability against HSV infection, and the highest neutralizing activity was associated with human IgG1 (murine IgG2a) [47,48]. We detected a significant decrease in IgG2a and IgG2b but not in IgG1 in the STAT4^−/−^ mice. Previously it was shown that murine IgG2a and IgG2b subtypes are able to fix complement [49]. These changes in the IgG subtypes in the STAT4^−/−^ mice suggest that these mice have less complement than wild-type control mice [49].

STAT4 responses did not appear to be involved in protection since mock-vaccinated STAT4^−/−^ mice were not more susceptible to lethal ocular HSV-1 infection than mock-vaccinated BALB/c mice. Mock-vaccinated STAT4^−/−^ mice had increased virus titers in their eyes on days 2 and 3 PI. However, this higher level of viral replication did not alter eye disease or survival in these mice, and it is likely that this increased viral replication was due to decreased T_H_1 responses. Previously we showed that an increase in IL-2 responses in the eye correlated with an increase in protection from eye disease in vaccinated mice [36,50]. The seemingly apparent lack of effect of STAT4^−/−^ in HSV-1 infection most likely indicates immune compensation orchestrated by STAT4-independent pathways [42] or differences in response from the triggering antigen as seen during influenza infection of STAT4^−/−^ mice [51] and in autoimmune diabetes [52]. In contrast to this study, it was previously shown that STAT4^−/−^ mice that were ocularly infected with the RE strain of HSV-1 had exacerbated eye disease when compared with control BALB/c mice [53]. The discrepancy between this study and our own may be related to the use of different virus strains or the methods used to measure protection.

In summary, the results presented here strongly suggest that STAT4 responses are not essential for vaccine-induced neutralizing antibody titers against HSV-1 or vaccine-induced protection against lethal HSV-1 infection. However, STAT4 responses were involved in induction of Ig subtype switching in the sera of ocularly infected mice. Taken together, our results suggest that enhanced T_H_1 responses are more effective in clearing HSV-1 infection, a result that mimics our previous vaccine studies [36,50].
